# Progress in Neurology

**Published:** 1898-07-09

**Authors:** 


### Progress in Neurology.

(*Continued from page* 238.)

**Treatment of Tabes.**—The suspension treatment of cases of locomotor ataxy has recently been experimentally investigated by De la Tourette and Chipault.[9] They find that suspension by Sayre's apparatus rarely increases the length of the spine by one centimetre, and has no effect at all on the length of the spinal cord; and probably on the living subject the perivertebral muscles prevent the weight of the lower limbs from having even this elongating effect. They find, however, that flexion of the spine produces a real measurable elongation of the cord. Experiments showed that the anterior wall of the vertebral canal was elongated by flexion to an extent varying from 3·1 to 4·3 cm. Measurements of the intradural elements showed that they too elongated, though to a less extent, on an average 1·8 cm., which was divided between the cord itself and the cauda equina, the cord stretching ·8 cm., and the cauda 1 cm. The various segments of the cord were differently affected, the maximum elongation was of the region between the twelfth dorsal and the first lumbar nerve. This method of flexion is applied by placing the patient in a sitting posture on a narrow table with fixed pelvis and forcibly flexing the trunk by means of a collar attached to the neck and shoulders. In the majority of cases the force used varied between 60 to 80 kilos. The sensation becomes, after a very short period, acutely painful, but after a few applications the patient is able to bear the operation fairly comfortably. The sitting should not last more than 8 to 10 minutes. On comparison of the results by forcible flexion with those obtained by suspension, it is found that by the latter plan decided amelioration of symptoms was obtained in 20 to 25 per cent., and slight improvement in another 30 to 35 per cent., whilst no benefit was found in 35 to 40 per cent. Of the patients treated by flexion, 46 per cent. were much improved in all their symptoms, and more especially as regards inco-ordination, pains, crises, anæsthesia, and retention of urine. Incontinence of urine was not so favourably influenced. Of the rest, in a large number one or two symptoms were benefited, whilst in 25 per cent. only no improvement was found—in contrast to the 35 to 40 per cent. of recorded failures after suspension. The cases treated by flexion were selected, both this and the suspension process being contra-indicated in very slowly progressing, very acute or very late cases.

Goldscheider[10] says that cases of locomotor ataxy are often benefited by exercise treatment, which was introduced by Fraenkel, whereby the muscular sense is re-educated. A chair may be inverted over the end of the bed, and the patient can then exercise himself by touching the cross-bars or by putting the feet in between them. The movements are first made with the eyes open, afterwards with the eyes shut. Ample periods of rest must be allowed so as not to produce fatigue, otherwise an exhaustion extending over several days may result. Even in advanced cases such improvement may occur that the patient may be able to walk again. Some patients do not improve, and sometimes the exercises have to be given up owing to the pains which are apparently induced by them. Hirschberg[11] has also treated nine cases of tabes by Fraenkel's method. The movements were practised for half an hour daily at first, and this time was gradually increased to one hour. The ataxy was considerably improved in every case, and concomitantly with this the patients were also subjectively improved. The contra-indications to the method of treatment are tabetic joint-lesions; blind patients find no benefit whatever. Eulenberg[12] also highly commends Fraenkel's treatment of ataxy by compensatory exercises.

Frenkel[13] finds that when ataxia is distinctly present in cases of tabes, disturbances in the perception of passive movements can always be found. In cases of tabes in which the most careful investigation fails to find evidences of ataxia, the degree of perception of passive movement varies; it may be normal, or the perception may be delayed. Important disturbances of sensation from the joints may be found in the toes or ankles without the presence of ataxia; but if such disturbances are found in the knee or hip, the presence of ataxia may always be demonstrated. When Romberg's sign is present the cutaneous sensibility of the soles is much altered; or, in addition to this, anomalies of sensation from the joints in the toes or from the ankle or knee-joint may be found. Romberg's sign without some disturbance of sensation does not exist in tabes.

[9] De la Tourette and Chipault, Gaz. des Hôpitaux, 1897. [10] Goldscheider, Deut. Med. Wochen, Nos. 4 and 5, 1898. [11] Hirschberg, Archiv. de Neurol. [12] Eulenberg, Deut. Med. Wochen, Oct. 28, 1897. [13] Frenkel, Neurol. Centralblatt, Nos. 15 and 16, 1897.

